# Routes of the Upper Branch of the Atlantic Meridional Overturning Circulation according to an Ocean State Estimate

**DOI:** 10.1029/2020GL089137

**Published:** 2020-09-14

**Authors:** Louise Rousselet, Paola Cessi, Gael Forget

**Affiliations:** ^1^ Scripps Institution of Oceanography University of California, San Diego La Jolla CA USA; ^2^ Department of Earth, Atmospheric and Planetary Sciences Massachusetts Institute of Technology Cambridge MA USA

**Keywords:** meridional overturning circulation, Lagrangian ocean analysis

## Abstract

The origins of the upper branch of the Atlantic meridional overturning circulation (AMOC) are traced with backward‐in‐time Lagrangian trajectories, quantifying the partition of volume transport between different routes of entry from the Indo‐Pacific into the Atlantic. Particles are advected by the velocity field from a recent release of “Estimating the Circulation and Climate of the Ocean” (ECCOv4). This global time‐variable velocity field is a dynamically consistent interpolation of over 1 billion oceanographic observations collected between 1992 and 2015. Of the 13.6 Sverdrups (1 Sv = 10^6^ m^3^/s) flowing northward across 6°S, 15% enters the Atlantic from Drake Passage, 35% enters from the straits between Asia and Australia (Indonesian Throughflow), and 49% comes from the region south of Australia (Tasman Leakage). Because of blending in the Agulhas region, water mass properties in the South Atlantic are not a good indicator of origin.

## Key Points

- The upper limb of the meridional overturning circulation originates primarily from the Tasman Leakage and the Indonesian Throughflow
- Ninety‐seven percent of the upper limb of the meridional overturning circulation enters the Atlantic from the Agulhas region
- Because of blending in the Agulhas region, water mass properties in the South Atlantic are not a good indicator of origin

## 1 Introduction

The meridional overturning circulation (MOC) of the Ocean is a key component of Earth's climate system. The Atlantic sector MOC transports heat northward at all latitudes (Hsiung, 1985), modulating the position of the Intertropical Convergence Zone (Kang et al., 2008).

**Figure 1:**
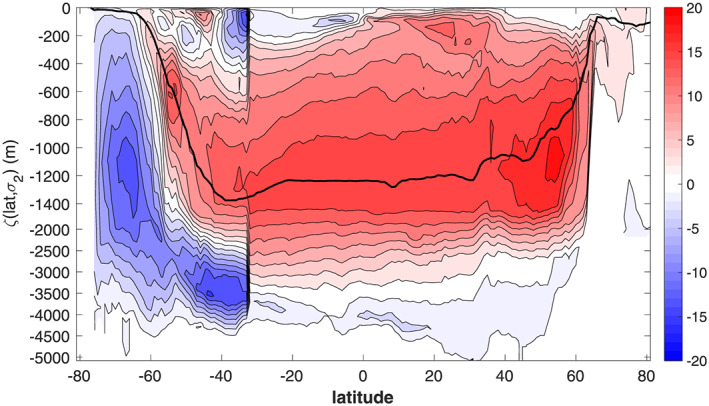
Residual meridional overturning circulation vertically integrated above surfaces of constant *σ*_2_, then time averaged and zonally integrated in the Southern Ocean south of 37°S, and in the Atlantic sector north of 37°S, as a function of latitude (abscissa) and *σ*_2_ (ordinate). The ordinate is remapped into a depth‐like coordinate *ζ*(latitude, *σ*_2_) which represents the time‐averaged and zonally averaged (over all longitudes) depth of each *σ*_2_ surface (expression (2) in the supporting information). The ECCOv4 (release 3) horizontal velocity (Eulerian + bolus), temperature, and salinity reanalysis fields are used. Positive values (red) indicate clockwise circulation. The contour interval is 2 Sv (1 Sv = 10^6^ m^3^/s). The thick black line marks $\zeta (\text{latitude},\sigma_{2}=36.6\;{\text{kg/m}}^{3})$, which approximately divides the upper and lower branches of the residual MOC.

The MOC includes northward flow of intermediate and upper waters from the Southern Ocean into the Atlantic, which are eventually transformed into North Atlantic Deep Water (NADW) in the Labrador and Nordic Seas. NADW then flows southward at depth upwelling in the Southern Ocean to close the mid‐depth cell (red contours in Figure 1). An equivalent mid‐depth cell is absent in the Indo‐Pacific sector (Cessi, 2019).

Water that has upwelled from the lower, southward branch of the mid‐depth cell in the Indo‐Pacific sector (south of 30°S) can return to the North Atlantic through two pathways: the warm route, that is, westward and northward around the tip of South Africa (Gordon, 1986), or the cold route, that is, eastward and northward around Drake Passage (Rintoul, 1991). The quantitative contributions of these two routes differ among estimates, but this partition is important for the transport of heat and freshwater into the Atlantic. Water that enters the South Atlantic through the warm route is warm and salty, while that entering through the cold route is fresh and cold. Many model simulations have shown that if the cold route prevails, then the MOC is robust to freshwater perturbation in the high latitudes of the North Atlantic and Arctic. Vice versa, if the exchange is mostly via the warm route, then North Atlantic freshwater perturbations shut down the MOC (Beal et al., 2011; de Vries & Weber, 2005; Drijfhout et al., 2011).

Several observational and numerical studies have estimated the relative contribution of the two routes. Most observational studies support the cold water route (Macdonald, 1998; Schmitz, 1995; Sloyan & Rintoul, 2001; Talley, 2013), while most numerical studies and one observational analysis favor the warm water route (Cessi & Jones, 2017; Donners & Drijfhout, 2004; Holfort & Siedler, 2001; Rodrigues et al., 2010; Rühs et al., 2019; Speich et al., 2001, 2007). Donners and Drijfhout (2004) illustrate the difficulty of establishing the origin of the MOC's upper branch using inverse models with sparse observations at hydrographic sections: This method applied to the output of an eddy‐resolving computation leads to a qualitatively different partition between routes than that obtained with Lagrangian analysis.

A major difficulty with identifying routes of the MOC is that the interbasin connection is mediated by zonal currents in the Southern Ocean, which are dominated by the zonal transport of the Antarctic Circumpolar Current (ACC) (Forget & Ferreira, 2019). The ACC transports about 50 Sv (1 Sv = 10^6^ m^3^/s) in the top 1,000 m of the water column, while the upper branch of the MOC carries about 14 Sv, a small fraction of the zonal circumpolar transport (Cessi, 2019). Another major difficulty is the paucity of trajectory measurements in the Southern Hemisphere ocean (Bower et al., 2019).

To overcome these difficulties, we quantify the pathways of upper and intermediate water from the Indo‐Pacific to the Atlantic using Lagrangian analysis. The method consists of tracking particle trajectories backward in time from an “exit” section in the South Atlantic (here 6°S) to specific “entry” sections. The origin of trajectories is identified by the backward‐in‐time first passage through one of the entry sections. Mass transport is quantified by initially populating the exit section with a large number of particles whose concentration is proportional to the local transport. Each particle carries a small amount of transport that is conserved following the trajectory because the velocity vector field conserves mass (volume). This type of calculations has been successfully performed using velocity fields from ocean general circulation models (Döös, 1995; Rühs et al., 2019; Speich et al., 2001), but not with global observations. To estimate transport with Lagrangian trajectories, it is necessary to have transport interpolated over a global grid, constrained by observations, while strictly conserving mass. A product that satisfies these requirements is the three‐dimensional, time‐variable, incompressible velocity from “Estimating the Circulation and Climate of the Ocean” (ECCOv4) (Forget, Campin, et al., 2015; Fukumori et al., 2017).

The modern measure of the MOC is given in terms of the “residual” transport within isopycnal layers, rather than depth layers. The residual overturning circulation measures the (potential) density transport, rather than the volume transport: It is more meaningfully associated with the transport of tracers than is the overturning in depth coordinates (Young, 2012). The residual overturning circulation captures the transport effected not only by the average meridional velocity but also by waves, eddies, and gyres with zero time‐averaged or zonally averaged velocity.

In models that do not resolve mesoscale processes, the eddy flux of tracers is parametrized as isopycnal diffusion and a “bolus” velocity related to the slope of isopycnals (Gent & McWilliams, 1990; Griffies, 1998; Redi, 1982). In these models the appropriate way to calculate the residual transport of the MOC is to use the sum of Eulerian plus bolus velocities, integrated over density layers rather than depth layers. In Figure 1, the time‐averaged and longitudinally integrated MOC is calculated in density coordinates and latitude, using *σ*_2_, that is, potential density referred to 2,000 decibars, as the density coordinate. Accordingly, the velocity used to calculate Lagrangian trajectories is the sum of Eulerian plus bolus velocity.

## 2 Calculation of Lagrangian Trajectories

The volume transports used in the calculation of Lagrangian trajectories are the monthly climatology available in release 3 of ECCOv4 on the native model grid at 1° horizontal resolution (the *Lat‐Lon‐Cap*‐90 grid as defined in Forget, Campin, et al., 2015) and with 50 vertical levels (Forget, Campin, et al., 2015; Fukumori et al., 2017). These transports derive from the dynamically consistent assimilation of over 1 billion observations for the period 1992–2015 into a primitive‐equations ocean‐sea‐ice model that satisfies exact conservation laws for mass, momentum, temperature, salinity, and sea‐ice.

The assimilated data consist of satellite products (including along‐track altimetry, mean dynamic topography, remotely sensed ocean bottom pressure, sea‐surface temperature, sea‐ice concentration, and surface salinity), and temperature and salinity profiles collected in situ (including from all Argo floats). To minimize misfit between the model and the observations, the following model parameters are optimized (Forget, Campin, et al., 2015; Fukumori et al., 2017): initial conditions in January 1992, air‐sea interactions throughout 1992–2015, diapycnal and isopycnal tracer diffusion rates, and the parameterized advective effect of mesoscale eddies (Forget, Ferreira, & Liang, 2015; Gent & McWilliams, 1990). Because the optimization uses the adjoint and forward versions of the model, for 50 iterations, the adjustment of the control parameters is beneficial on a time scale of hundreds of years, even though the data record is only 24 years long (Forget, Ferreira, & Liang, 2015).

Many studies have looked at various measures to assess the ECCOv4 estimate: These collectively affirm its value for understanding both the Ocean climatology and its variability from observations (Forget, Campin, et al., 2015; Forget & Ferreira, 2019; Forget & Ponte, 2015; Fukumori et al., 2017; Jackson et al., 2019). The estimate of the MOC according to ECCOv4 is in broad agreement with several independent (i.e., not assimilated) estimates (see Table 1 in Cessi, 2019). The ACC transport at Drake Passage (DP) in ECCOv4 is 155 Sv which is intermediate between the two recent independent “in situ” estimates of Cunningham et al. (2003) (134 ± 11 Sv) and Donohue et al. (2016) (173 ± 11 Sv) and in the middle of the reanalyses ensemble compiled by Uotila et al. (2019) (152 Sv [±19.2 Sv]). ECCOv4 accurately reproduces the transport of properties, but the limited resolution might distribute it on scales larger than in nature.

To determine Lagrangian trajectories, the ECCOv4 climatological monthly volume transports are interpolated in space and time using an incompressibility‐preserving algorithm (Blanke & Raynaud, 1997; Döös, 1995) and advected backward in time for 2011 years (more details in the supporting information, SI hereafter) (Campin et al., 2019). Because these climatological transports are 1‐year periodic, the annual cycle can be repeated as many times as needed, without introducing discontinuities that artificially alter the trajectories. Particles are initialized every month for the first year (for a total of 63,482 particles) at 6°S in the South Atlantic, at depths above the $\sigma_{2}=36.6\;{\text{kg/m}}^{3}$ surface: This defines the “exit” section. The $\sigma_{2}=36.6$ kg/m^3^ surface marks the lower boundary of the upper, northward branch of the MOC at 6°S. Each particle carries about 2 × 10^−4^ Sv so that the number of particles per each grid face on the section is proportional to the transport across that grid face (the exact transport of each particle is recorded). Because the velocity vector is exactly incompressible, the transport of each particle is conserved following the trajectory (Blanke & Raynaud, 1997; Döös, 1995). This conservation can be used to quantify the transport through different “entry” sections, determining the origin of waters that feed the upper branch of the MOC. The place and time of origin is defined as being the first passage (backward in time) from the exit to one of the four entry sections.

**Figure 2:**
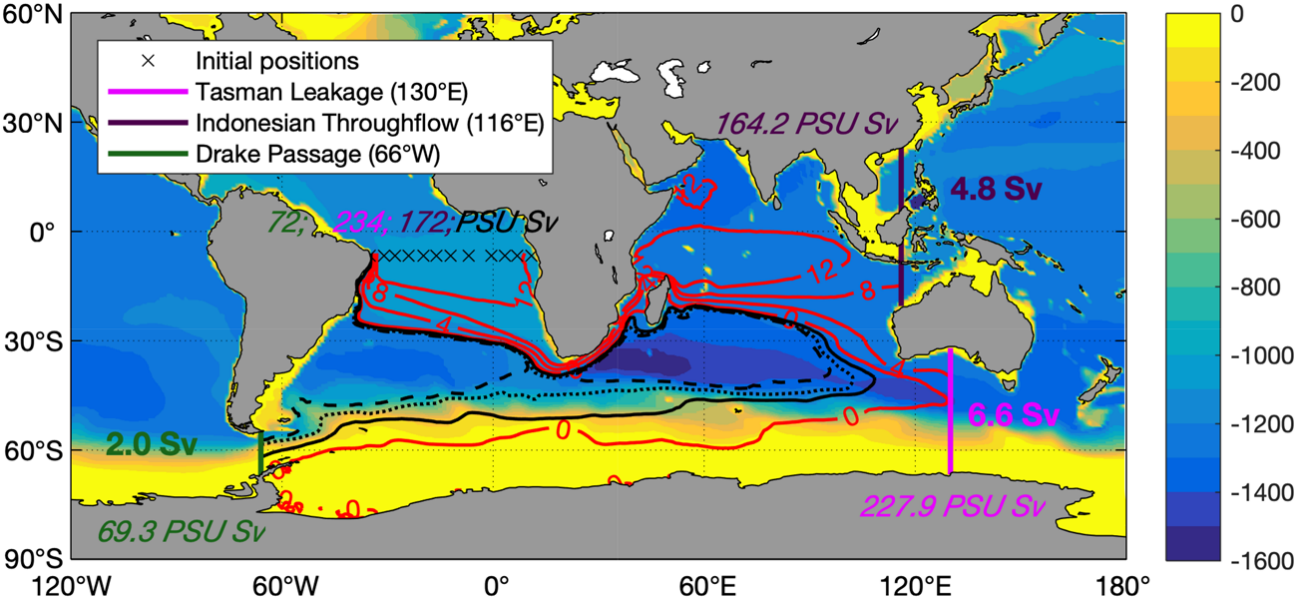
Transport stream function calculated from particle trajectories (contours): Red contours are 4 Sv apart; the black solid contour is −1 Sv, the black dotted contour is −1.5 Sv, and the black dashed contour is −1.75 Sv. The color shading shows the depth (in m) of the time‐averaged $\sigma_{2}=36.6\;{\text{kg/m}}^{3}$. The exit section at 6°S is marked by black x, and the entry sections are at DP (66°W green), at TL (130°E magenta), and IT (116°E dark purple). The volume (in Sv) and salinity transports (in PSU Sv) at each section are marked in the corresponding color. The volume transport at the exit section is 13.6 Sv. After 2011 years there are 0.04 Sv still recirculating in the region bounded by the four sections, and 0.2 Sv have entered 6°S from the North below $\sigma_{2}=36.6\;{\text{kg/m}}^{3}$.

The entry sections at which we measure the first passage are DP (at 66°W); Indonesian Throughflow (IT, at 116°E); Tasman Leakage (TL, at 116°E); and South Atlantic at 6°S for depths below the $\sigma_{2}=36.6$ kg/m^3^ surface, that is, in the lower limb of the MOC (cf. Figure 2). The northward transport across the exit section at 6°S is 13.64 Sv. The particles are followed for 2011 years, after which all but 0.3% have moved through one of the entry sections. The region encompassed by the entry and exit sections has a net evaporation of 0.44 Sv, but our procedure does not allow particles to exit or enter the air‐sea boundary: Instead, particles that try to escape the surface are reinjected into the first model level. The computation was repeated for 512 years with double the number of particles (126,964), and the transport values at the exit region differ at most by 0.2% over that time from those with 63,482 particles. We present the result with 63,482 particles, which we trust to be robust. By convention, the particle trajectories and their transport are described as forward in time hereafter.

## 3 Routes of the Upper Limb of the MOC

The MOC upper limb pathways are quantified by considering the subsets of trajectories connecting the exit section (6°S) with each of the entry sections. Figure 2 shows the transport stream function (contours) obtained by summing vertically the particles in bins of 1° × 1°, weighted by their transport (magnitude and sign). The color shading shows the depth of the $\sigma_{2}=36.6\;{\text{kg/m}}^{3}$ surface, averaged over the 24‐year period covered by ECCOv4. The transport stream function flows around the edge of the Southern Ocean supergyre, whose shape is highlighted by the depth contours of $\sigma_{2}=36.6\;{\text{kg/m}}^{3}$ (Speich et al., 2002, 2001, 2007). All trajectories converge to the region separating the subtropical and tropical gyres in the South Atlantic before reaching 6°S. The net transport at the exit section is composed of

DP: 2.0 Sv across DP at 66°W;
IT: 4.8 Sv across the IT at 116°E;
TL: 6.6 Sv at 130°E, south of Australia (TL); and
6°S: 0.2 Sv at 6°S flowing southward for $\sigma_{2}>36.6\;{\text{kg}/m}^{3}$.

**Figure 3:**
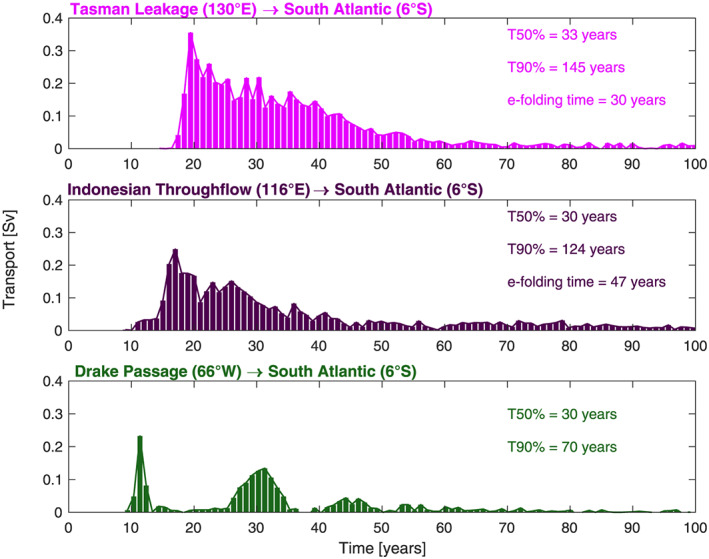
Distribution of transit times from the exit section at 6°S to each entry section weighted by transport. The inset also shows the median, the 90th percentile, and the e‐folding time scales fitted to the distributions between 9 and 150 years.

Trajectories that enter through DP flow around the edge of the subtropical gyres of the South Atlantic and South Indian Oceans before moving northward across the section at 6°S. Thus, a large fraction of the particles entering DP (80% or 1.6 Sv) subsequently goes through the Agulhas current region. This pathway has been termed the “indirect cold route” (Gordon et al., 1992) and has a typical transit time longer than 25 years, shown by the secondary peaks in the distribution on the bottom panel of Figure 3. The interpeak intervals correspond to the transit times around the supergyre. The direct cold route, which avoids the Agulhas Current, is a small fraction (20% or 0.4 Sv), with typical transit times of 12 years.

Most trajectories come through TL (e.g., red contours 0 and 4 Sv in Figure 2) and IT (e.g., red contours 8 and 12 Sv in Figure 2), all steered by the flow around the outer edge of the supergyre. TL carries more transport (6.6 Sv) than IT (4.8 Sv), while the opposite is true in the previous Lagrangian analysis of Speich et al. (2001) using a 2°‐resolution ocean model nudged to the climatological temperature and salinity of the Levitus (1982) atlas. Our estimate of the median transit time (see Figure 3) is 30 years for DP and IT, and 33 years for TL. All median transit times are shorter than those of Speich et al. (2001), who found 52 years for DP and IT, and 81 years for TL. In all cases, there are long tails in the transit‐time distributions, indicating extensive recirculations. Qu et al. (2019) document an increase in the Agulhas Leakage and TL of 2 Sv/decade, and an IT increase of 0.6 Sv/decade, all associated with a spin‐up of the supergyre during the 24 years of ECCOv4 assimilation: This spin‐up could explain our shorter transit times.

The total transport entering the South Atlantic from the east at 22°E carries 97% of the transport, while DP through the direct cold route carries 3% of the MOC. These results should be compared with Speich et al. (2001) who found an 87–13% split. The increases in Agulhas, TL, and IT transports, documented for the ECCOv4 assimilation period, might account for the differences (Qu et al., 2019).

The recent analysis of an eddy‐resolving simulation, unconstrained by observations, finds a 60–40% split between these two routes, that is, 10 times what we find for the direct cold route (Rühs et al., 2019). One reason for this difference is that ECCOv4 has a stronger climatological eastward transport of the ACC at DP (155 Sv) than the eddy‐resolving, non‐assimilative model simulation of Rühs et al. (2019) (116 Sv). Trajectories crossing DP are swept away more efficiently and more realistically by the stronger zonal flow. The large‐scale shape and transport of the supergyre also differ in the two estimates: These upper‐ocean properties are well constrained by observations in ECCOv4, as is the Agulhas leakage transport (Qu et al., 2019).

## 4 Thermodynamic Properties of the Upper Limb of the MOC

**Figure 4:**
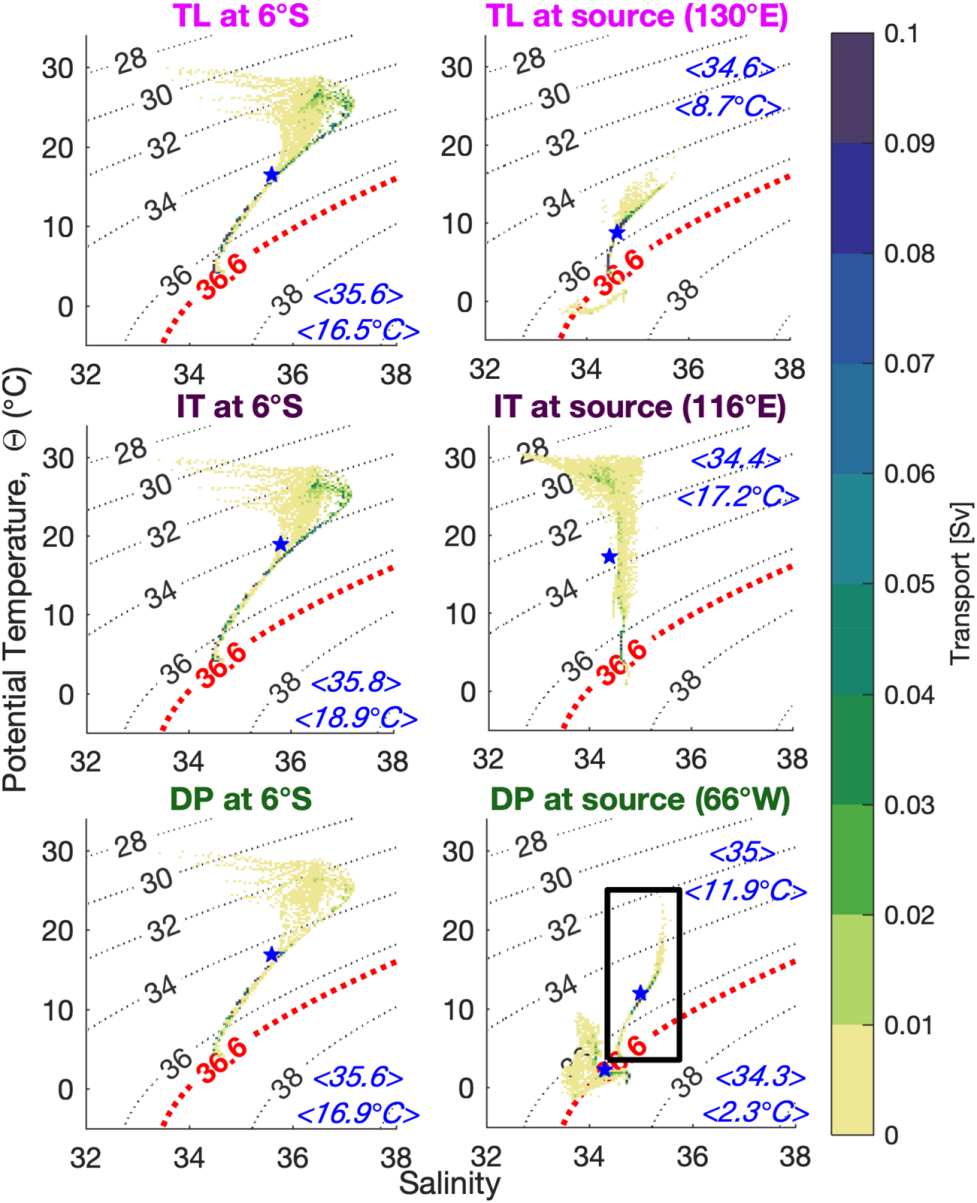
Transports (colorbar, in Sv) binned in potential temperature/salinity space following trajectories from the entry (right) to exit (left) sections. Trajectories entering from TL are in the top row, from IT in the middle row, and from DP in the bottom row. The boxed inset in the bottom right panel is for all particles crossing the Agulhas section at 22°E. This section carries 97% of the exit transport. Dashed lines show contours of constant *σ*_2_. Each blue star marks the average value of the underlying distribution, denoted in blue by ⟨*θ*⟩ (in °C) and ⟨*S*⟩ (in PSU).

The thermodynamic characteristics at the entry regions differ markedly between DP, IT, and TL. The right panels in Figure 4 show the potential temperature‐salinity (*θ*‐*S*) volumetric diagram (colored points, weighted by transport), for the particles at three entry regions, plus at the Agulhas section at 22°E (boxed inset in bottom right panel). The water at DP is cold and fresh; the water at IT is warm and slightly saltier; and the water at TL is intermediate in temperature between DP and IT and saltier. TL water includes a fraction which shares properties originating at DP, that is, cold and relatively fresh: This water is associated with the outer streamlines (positive near zero values in Figure 2), which flow eastward in the southern region of the ACC and then turn around to enter the TL section.

As in the original “warm‐cold route” nomenclature (Gordon, 1986), there is a substantial difference in temperature between the waters originating in DP and those originating in IT and TL, while the distinction in salinity is less pronounced. By the time the particles reach 6°S, the differences between these characteristics are erased, and the three groups are undistinguishable in *θ*‐*S* space, as found in Rühs et al. (2019). This is because all trajectories eventually merge into the narrow South Equatorial Current and North Brazil Current before reaching 6°S, facilitating properties' exchanges. Furthermore, IT, TL, and indirect‐route DP waters are all squeezed into the narrow Agulhas Current system, from which they emerge as a single water mass in the South Atlantic, with a tight *θ*‐*S* relation at 22°E (the boxed inset in the bottom right panel of Figure 4), dominated by a blend of TL and IT properties.

At the 30°S and 22°E (Agulhas) sections, 62% of the transport has *σ*_2_ < 35.7 (upper water), and 38% has 35.7 ≤ *σ*_2_ ≤ 36.8 (intermediate water), that is, a larger preponderance of surface water over intermediate water compared to the 1°‐resolution ocean model nudged to the climatological temperature and salinity of the World Ocean Atlas (Locarnini et al., 2013; Zweng et al., 2013) by Lee et al. (2019). Upper (intermediate) water are thought to originate from the warm (cold) route, but the fractions in *σ*_2_ space above give a different measure of the origin of waters compared to the Lagrangian analysis.

The bulk of particles arriving at 6°S have a salinity which is larger than any of the water masses at their sections of origin, or at 22°E. The increase in Lagrangian transport of salinity between the entry and exit sections is 16.6 PSU Sv (cf. the color‐coded salinity transports marked in Figure 2), which requires evaporation of 0.46 Sv at an average surface salinity of 36 PSU. This is consistent with the surface freshwater loss of 0.44 Sv estimated by ECCOv4 in this region.

While particles become saltier from their region of origin at DP, IT, and TL, the particles from DP and TL warm up sufficiently to overcome the density increase due to salinification. Thus, with the exception of particles originating in the IT, the particles at 6°S are less dense than at their origin. The particles originating at the IT experience a general cooling as they pass through the Agulhas Current system, only to be warmed up again in the South Atlantic. Typical trajectories from the entry sections of TL, IT, and DP (including both direct and indirect routes) to the exit section at 6°S are shown in the supporting information. The animations provide a visualization of time scales, pathways, and thermohaline transformations along the trajectories.

## 5 Conclusions

We evaluate the routes and thermohaline properties of the upper branch of the MOC with a Lagrangian analysis using the three‐dimensional velocity of ECCOv4, a state estimate which is a close fit to most available global data constraints. We find that the upper branch of the MOC receives its transport primarily from the region south of Australia (TL), followed by a close second contribution from the IT, and a distant third contribution from DP.

Eighty percent of the particle transport through 6°S originating from DP goes through the indirect cold route. The multimodal transit‐time distribution for particles originating at DP illustrates the difference between the direct and indirect cold routes. We find typical transit times shorter than a Lagrangian analysis constrained by the Levitus (1982) climatology, possibly due to the spin‐up of the supergyre documented by Qu et al. (2019).

The common path through the Agulhas region results in a loss of identity in *θ*‐*S* and density space, casting doubts on the ability to recognize the origin of this transport through comparative analysis of water masses. The contributions from DP, TL, and IT have already the same thermohaline properties when entering the South Atlantic. While it is not possible to distinguish the origin of water using water mass characteristics, the resulting blend is dominated by the contribution of the largest routes, that is, by TL and IT: the pathways of the MOC's upper limb matter.

## Supporting information



Supporting Information S1

Movie S1

Movie S2

## Data Availability

The monthly climatology used in this study is provided by the ECCO Consortium and is freely available online (https://ecco.jpl.nasa.gov/drive/files/Version4/Release3/). The Lagrangian software (FLT package) employed to compute the trajectories is available in the GitHub repository of the MITgcm suite (https://github.com/MITgcm/MITgcm/tree/master/pkg/flt). The customized code of the FLT package developed for this study will be soon uploaded on the GitHub platform.
