# Supplementary material for: Routes of the Upper Branch of the Atlantic Meridional Overturning Circulation according to an Ocean State Estimate
Source: Geophys Res Lett. 2020 Sep 14;47(18):e2020GL089137. doi: 10.1029/2020GL089137 (PMC7757194; doi:10.1029/2020GL089137)
Supplement: Supplementary file 1 — Supporting Information S1 [file GRL-47-e2020GL089137-s001.pdf]

Supporting Information for "Routes of the upper branch of the Atlantic Meridional Overturning Circulation according to an ocean state estimate"

Louise Rousselet[1], Paola Cessi[1] *, Gael Forget[2]

[1]Scripps Institution of Oceanography, University of California, San Diego, USA

[2]Dept. of Earth, Atmospheric and Planetary Sciences, Massachusetts Institute of Technology, Cambridge, MA, USA

Contents of this file

1. Text S1 to S6
2. Figure S1 to S2

Additional Supporting Information (Files uploaded separately)

1. Captions for Movies S1 to S2

*Paola Cessi, Scripps Institution of Oceanography,
University of California, San Diego,
La Jolla, CA 92039-0213, USA

Introduction

The residual overturning circulation is shown as a function of σ_2. A description of the algorithm and software used for the Lagrangian trajectories is provided, together with two animations of four typical particle trajectories.

Residual overturning circulation definitions

The residual overturning circulation differs from its Eulerian counterpart in that the transport is integrated at fixed density rather than at fixed depth. The advantage of this approach is that density transport includes the components that have zero average (the average might be in time, space or both), such as waves, eddies and large-scale gyres. The zonally integrated and time-averaged residual overturning circulation, $\psi^\dagger$, is defined as

$$\psi^\dagger(latitude, \sigma_2) \equiv \overline{\int_{x_w}^{x_e} dx \int_{-H}^{-h(x,latitude,\sigma_2,t)} (v + v_{GM})\, dz} \tag{1}$$

where $v + v_{GM}$ is the Eulerian plus bolus meridional velocity, $x_{w,e}$ are the longitudes of the domain boundaries, $z = -h(x, latitude, \sigma_2, t)$ is the depth of each σ_2-surface, $z = -H$ is the ocean bottom, and the overline represents time average.

For density to be useful a vertical coordinate it needs to be monotonic. Thus we use σ_2 which is stably stratified throughout the water column (small regions of non-monotonicity are acceptable). The disadvantage is that σ_2 as a vertical coordinate is less familiar and intuitive than depth. One way to overcome this disadvantage is to remap σ_2 in terms of the average depth of each σ_2-surface, $\zeta(latitude, \sigma_2)$ (Young, 2012). We define

$$\zeta(latitude, \sigma_2) \equiv \langle \overline{\int_{-h(x,latitude,\sigma_2,t)}^{0} dz} \rangle\,, \tag{2}$$

where $\langle\ \rangle$ is the zonal average at all longitudes. This remapping allows to visualize $\psi^\dagger$ in terms of the depth-like coordinate, ζ, while preserving its dependence on σ_2. The

vertical coordinate in the residual overturning streamfunction of figure 1 in article is $\zeta(latitude, \sigma_2)$. Figure S.1 shows the same streamfunction using σ_2 as the vertical coordinate: the values of $\psi^\dagger$ are the same in both views, but the vertical stretching is different.

Particle-trajectory calculation

ECCOv4 provides the three-dimensional velocity field on a gridded mesh in latitude, longitude and depth for the 24 years of the assimilated period. We use the monthly climatological average, repeated for 2011 years over the annual period. The monthly climatology averages each field over the days in each month, and each of the twelve months over the 24 years of the assimilation. The twelve monthly values for each field are interpolated linearly in time to construct a signal which is one-year-periodic. In this way, the fields are exactly periodic over 365 days and there are no jumps between months or when a new year starts, but the interannual variability is lost.

In order to preserve the conservation properties of the gridded ECCOv4 fields, and in particular the incompressibility of the velocity, it is important to use the fields on the native curvilinear grid (the interpolation and average in time do not affect incompressibility). The global domain of ECCOv4 is decomposed in 13 tiles, and particle trajectories must be exchanged across the tiles' boundaries (Forget et al., 2015). To our knowledge, the only code that is capable of seamlessly exchanging particles across tiles on the three dimensional ECCOv4 curvilinear grid is the FLT package within the MITgcm suite and this is what we used (Campin et al., 2019). Additionally, the FLT package is computationally efficient on the multi-processor, multi-node supercomputer available to us.

The spatial interpolation algorithm for the velocity field was modified according to Döös (1995), to *linearly* interpolate the *transport* associated with each velocity component on the staggered C-grid used by ECCOv4: in this way incompressibility of the velocity is guaranteed at every point along the particle trajectory. Because the observational constraints are applied to the *transports* in ECCOv4, the errors in the trajectories are minimized. In particular, with velocity incompressibility satisfied exactly, particles never reach the land points where the transport vanishes, and no particles are lost. Unlike Döös (1995), we use a fourth order Runge-Kutta scheme to time-step the trajectories, with a time-step of five hours; this introduces a small error in the time-integration, without violating incompressibility. We have tested that decreasing the time step to one hour introduces an average difference in the trajectory position of 1×10^{-3} degrees, at the cost of tripling the computational time.

Particle-trajectory animations

In figure S.2 and in the associated animations, four particles are tracked from their initial entry in one of the sections to the exit section at 6°S in the Atlantic. The particles are chosen as they best represent: (i) the average potential temperature and salinity at each entry section (i.e. ± 0.2 from mean θ°C and ± 0.1 PSU from mean salinity); (ii) the median transit times shown in Figure 2 (i.e. ± 15 years from T50%). Particle entering through Tasman Leakage and Indonesian Throughflow are shown respectively with a diamond and a circle. Both "direct" and "indirect" cold routes from Drake Passage are shown by two different trajectories (stars). In addition to the position, which is shown every 3 months, the color of the particle denotes its potential temperature or salinity at

that position, allowing to visualize the thermodynamic transformations occurring along each trajectory. The temperature and salinity variations over time (along trajectories) are also displayed in the bottom right inset. The bottom left legend indicates the arrival time of each particle at 6°S.

Movie S1. Positions of four particles along typical trajectories from the entry sections are shown every 3 months with different symbols: stars denote particles entering at DP, diamonds are for entry at TL, and dots are for entry at IT. The color of the symbols denotes salinity (in PSU). The salinity evolution for each particle is shown in the bottom right inset. The bottom left legend indicates the arrival time of each particle at 6°S.

Movie S2. Positions of four particles along typical trajectories from the entry sections are shown every 3 months with different symbols: stars denote particles entering at DP, diamonds are for entry at TL, and dots are for entry at IT. The color of the symbols denotes potential temperature (in °C). The temperature evolution for each particle is shown in the bottom right inset. The bottom left legend indicates the arrival time of each particle at 6°S.

References

Campin, J.-M., Heimbach, P., Losch, M., Forget, G., edhill3, Adcroft, A., … McRae, A. T. T. (2019, October). *Mitgcm/mitgcm: checkpoint67m.* Zenodo. Retrieved from https://doi.org/10.5281/zenodo.3492298 doi: 10.5281/zenodo.3492298

Döös, K. (1995). Interocean exchange of water masses. *J. Geophys. Res.*, *100*, 13499–13514.

Forget, G., Campin, J.-M., Heimbach, P., Hill, C. N., Ponte, R. M., & Wunsch, C.

(2015). ECCO version 4: an integrated framework for non-linear inverse modeling and global ocean state estimation. *Geoscientific Model Development*, *8*(10), 3071–3104. Retrieved from `http://www.geosci-model-dev.net/8/3071/2015/` doi: 10.5194/gmd-8-3071-2015

Young, W. R. (2012). An exact thickness-weighted average formulation of the Boussinesq equations. *J. Phys. Oceanogr.*, *42*(5), 692-707.

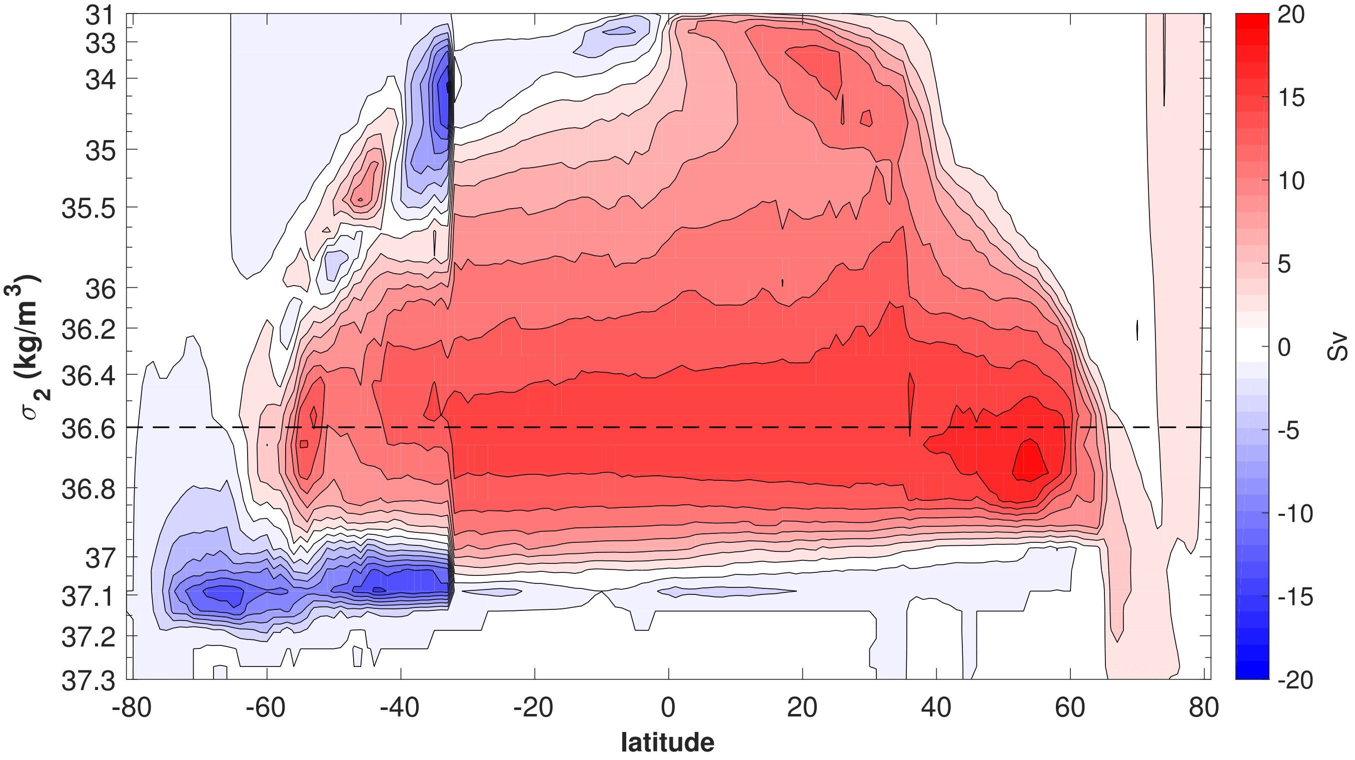

Figure S.1. Residual meridional overturning circulation vertically integrated above surfaces of constant σ_2, then time averaged and zonally integrated in the Southern Ocean south of 37°S, and in the Atlantic sector north of 37°S, as a function of latitude (abscissa) and σ_2 (ordinate). The ECCOv4 (release 3) horizontal velocity (Eulerian+bolus), temperature and salinity reanalysis fields are used. Positive values (red) indicate clockwise circulation. The contour interval is 2 Sv (1 Sv = 10^6 m^3/s). The dashed line marks σ_2=36.6 kg/m^3), which approximately divides the upper and lower branches of the residual MOC.

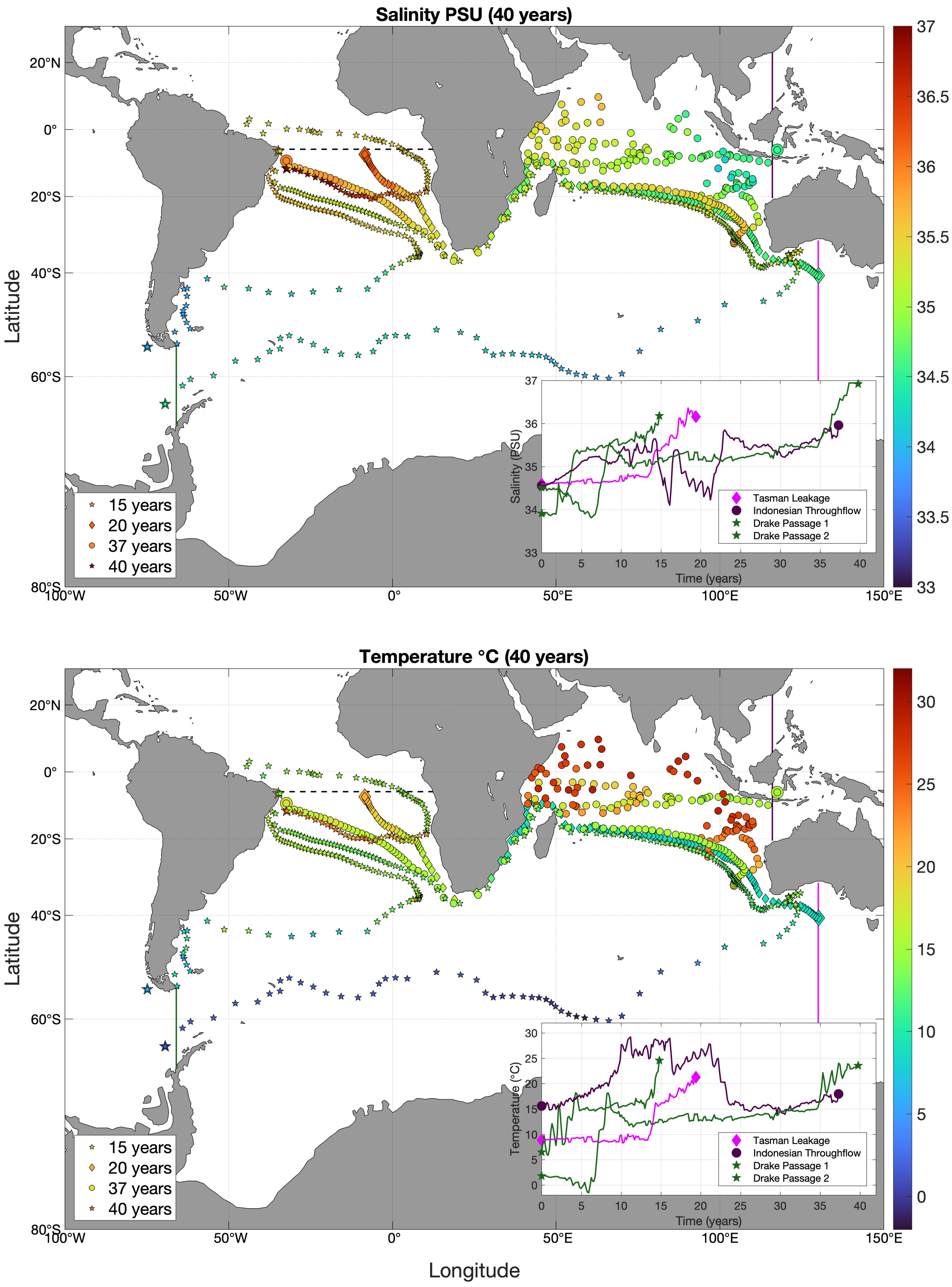

Figure S.2. Positions of four particles along typical trajectories from the entry sections are shown every 3 months with different symbols: stars denote particles entering at DP, diamonds are for entry at TL, and dots are for entry at IT. The color or the symbols denote salinity (in PSU) in the top panel and potential temperature in the bottom panel (in $^\circ$ C). The salinity and temperature evolution for each particle is shown in the bottom right inset. The bottom left legend indicates the arrival time of each particle at 6°S.
